# Gold Nanoparticle-Enhanced and Roll-to-Roll Nanoimprinted LSPR Platform for Detecting Interleukin-10

**DOI:** 10.3389/fchem.2020.00285

**Published:** 2020-05-26

**Authors:** Seung Hee Baek, Hyun Woo Song, Sunwoong Lee, Jung-Eun Kim, Yeo Hyang Kim, Jung-Sub Wi, Jong G. Ok, Jun Seok Park, Seonki Hong, Moon Kyu Kwak, Hye Jin Lee, Sung-Wook Nam

**Affiliations:** ^1^Department of Molecular Medicine, School of Medicine, Kyungpook National University, Daegu, South Korea; ^2^Department of Mechanical Engineering, School of Mechanical Engineering, Kyungpook National University, Daegu, South Korea; ^3^Department of Pediatrics, School of Medicine, Kyungpook National University, Daegu, South Korea; ^4^Center for Nano-Bio Measurement, Korea Research Institute of Standards and Science, Daejeon, South Korea; ^5^Department of Mechanical and Automotive Engineering, Seoul National University of Science and Technology, Seoul, South Korea; ^6^Department of Surgery, School of Medicine, Kyungpook National University, Daegu, South Korea; ^7^Department of Emerging Materials Science, Daegu Gyeongbuk Institute of Science and Technology (DGIST), Daegu, South Korea; ^8^Department of Chemistry and Green Nano Materials Research Center, Kyungpook National University, Daegu, South Korea

**Keywords:** Au nanocube, IL-10, Au LSPR strip, roll-to-roll, nanoarchitecture

## Abstract

Localized surface plasmon resonance (LSPR) is a powerful platform for detecting biomolecules including proteins, nucleotides, and vesicles. Here, we report a colloidal gold (Au) nanoparticle-based assay that enhances the LSPR signal of nanoimprinted Au strips. The binding of the colloidal Au nanoparticle on the Au strip causes a red-shift of the LSPR extinction peak, enabling the detection of interleukin-10 (IL-10) cytokine. For LSPR sensor fabrication, we employed a roll-to-roll nanoimprinting process to create nanograting structures on polyethylene terephthalate (PET) film. By the angled deposition of Au on the PET film, we demonstrated a double-bent Au structure with a strong LSPR extinction peak at ~760 nm. Using the Au LSPR sensor, we developed an enzyme-linked immunosorbent assay (ELISA) protocol by forming a sandwich structure of IL-10 capture antibody/IL-10/IL-10 detection antibody. To enhance the LSPR signal, we introduced colloidal Au nanocube (AuNC) to be cross-linked with IL-10 detection antibody for immunogold assay. Using IL-10 as a model protein, we successfully achieved nanomolar sensitivity. We confirmed that the shift of the extinction peak was improved by 450% due to plasmon coupling between AuNC and Au strip. We expect that the AuNC-assisted LSPR sensor platform can be utilized as a diagnostic tool by providing convenient and fast detection of the LSPR signal.

## Introduction

Localized surface plasmon resonance (LSPR) sensors can monitor a variety of binding events including protein-ligand interactions (McFarland and Van Duyne, 2003; Holzinger et al., 2014; Jang et al., 2014; Liu et al., 2018), DNA hybridizations (Storhoff et al., 2004; Sönnichsen et al., 2005; Baek et al., 2014), and biomarker interactions (Im et al., 2014; Xu et al., 2018; Bellassai et al., 2019; Wang et al., 2019). When binding events occur, the change in LSPR properties manifest as a shift of the extinction peak, which is easily measured by ultraviolet-visible spectroscopy (UV-vis) (Sepúlveda et al., 2009). The extinction behavior comprises both absorption and scattering events of the incident light by the LSPR sensor. Due to its simple and convenient characterization process, the LSPR sensor has been considered as a potential detection tool in enzyme-linked immunosorbent assays (ELISA) (Chen et al., 2011).

It is known that the extinction peak shift stems from the changes of the refractive index on the surface of the LSPR structure when analyte binds with the plasmonic nanostructures such as gold (Au) and silver (Ag) (Willet and Van Duyne, 2007; Hall et al., 2011). To improve the LSPR detection capability, we developed dual plasmonic structures by inducing plasmon coupling between colloidal Au nanocube (AuNC) and multi-bent Au nanostrip.

Recently, Wi et al. investigated a double bent Au structure on a flexible polyethylene terephthalate (PET) film and its effect on the sensitivity of LSPR properties, which enables the detection of proteins with femtogram-level sensitivity (Wi et al., 2017). In the report, the finite-difference time-domain (FDTD) simulations show that the folding of the Au structures accommodates the longer plasma oscillations in the confined structures, allowing for high sensitivity to the refractive index change of the surrounding media (Miller and Lazarides, 2005; Mayer and Hafner, 2011). Here, we present a roll-to-roll nanoimprint method to produce nano-architecture platforms such as nanoline or nanodot structures. In particular, Au deposition upon the nanograting platform results in the construction of a multi-bent Au structure with a strong extinction peak at ~760 nm. With this Au LSPR platform, we introduce a method of detecting interleukin-10 (IL-10), one of the cytokines involved in maintaining homeostasis of the immune system.

To apply the multi-bent Au structure for the ELISA protocol, we attempted to integrate the dual Au nanoparticle/Au strip structures into the benchtop microplate reader equipment. Specifically, we attached PET films having multi-bent Au structures to the bottom of a conventional 96-well microplate, which is then soaked in reagent solution. We performed an ELISA protocol using standard reagents including capture antibody, target analyte, and primary antibody. To detect IL-10 protein specifically bound to IL-10 antibody, we utilized the colloidal AuNC as an immunogold assay assistant in the ELISA protocol. As a result, we observed that AuNC enhances the peak shift by 450% compared to when AuNC is absent. Based on this dual Au LSPR assay platform, we succeeded in detecting IL-10 model protein in the nanomolar range.

## Materials and Methods

### Materials

The human IL-10 uncoated ELISA kit including IL-10 protein, IL-10 capture antibody, and IL-10 detection antibody was purchased from Invitrogen. Gold (III) chloride hydrate (HAuCl_4_), sodium borohydride (NaBH_4_), L-ascorbic acid, 11-mercaptoundecanoic acid (MUA), and bovine serum albumin (BSA) were purchased from Sigma-Aldrich. 1-Ethyl-3-(3-dimethylaminopropyl) carbodiimide hydrochloride (EDC) and N-hydroxysulfosuccinimide (NHSS) were purchased from Thermo Fisher Scientific Inc. Hexadecyltrimethylammonium bromide (CTAB) was purchased from Tokyo Chemical Industry Co., Ltd. Phosphate-buffered saline (PBS) for biofunctionalization of AuNC was purchased from Biosesang.

### Au LSPR Strip Fabrication

Au LSPR strip was fabricated using roll-to-roll nanoimprint lithography (Koo et al., 2016; Wi et al., 2017). First, nanograting structures were fabricated with polyurethane acrylate (PUA) on PET film using a 200 nm-pitch and 100 nm-height silicon master. Titanium (Ti) (thickness of 5 nm) and Au (thickness of 50 nm) were sequentially deposited on the fabricated nanograting structures by thermal evaporator (Ultech, EasyDEP-5).

### Synthesis of AuNC Colloid

AuNC were synthesized according to the previously reported seeding-growth method (Sau and Murphy, 2004; Wu et al., 2010; Jang et al., 2014). Seed nanoparticle solution was prepared by adding 250 μL of 0.01 M HAuCl_4_ and 600 μL of 0.01 M NaBH_4_ to 7.5 mL of 0.1 M CTAB, as described in Figure S1A. The solution was matured at 29°C for 1 h. For the growth solution, 6.4 mL of 0.1 M CTAB, 800 μL of 0.01 M HAuCl_4_ solution, 3.8 mL of 0.1 M L-ascorbic acid solution, and 20 μL of 10X-diluted seed solution were sequentially added to 32 mL DI water, as shown in Figures S1B,C. The resulting solution was incubated at 29°C overnight. We centrifugated the solution at 11,000 rpm for 6 min and resuspended it in DI water to remove excess CTAB.

### Immobilization of Detection Antibody on the Surface of Colloidal AuNC

To form a self-assembled monolayer (SAM) on AuNC surface, 10 μL of 10 mM MUA was added to 990 μL of AuNC solution prior to antibody attachment via EDC/NHSS cross-linking chemistry. The MUA-coated Au surfaces were reacted with 10 μL of 7.5 mM EDC and 1.5 mM NHSS in DI water at room temperature for 30 min. The resulting AuNC solution was left for 3 h at 29°C after the addition of IL-10 detection antibody. After the reaction, the antibody-immobilized AuNC solution was centrifugated at 11,000 rpm for 6 min and resuspended in PBS. The biofunctionalized AuNC colloid was characterized via bench-top microplate reader (Molecular Devices, SpectraMax).

### Immobilization of Capture Antibody on the Surface of Au LSPR Strip

A similar surface chemistry was used for immobilization of the IL-10 capture antibody on the Au LSPR strip. The Au LSPR strip was soaked in ethanolic MUA solution overnight. After the formation of SAM, 10 μL of 7.5 mM EDC and 1.5 mM NHSS in DI water were reacted upon the Au LSPR strip at room temperature for 30 min. The carboxylic acid terminated Au surface was kept for 3 h at 29°C after the addition of IL-10 capture antibody. The surface-modified Au LSPR strip was characterized by the microplate reader with a normal colorimetric scanning mode.

### Characterizations of AuNC and Au LSPR Strip

AuNC and Au LSPR strip were characterized by field emission scanning electron microscopy (FE-SEM, Hitachi SU8220) and field emission transmission electron microscopy (FE-TEM, FEI Titan G2 ChemiSTEM, Cs probe corrector) with energy-dispersive X-ray spectroscopy (EDX). For chemical analysis of AuNC, the sulfur (S) element present on the Au surface was analyzed using inducted coupled plasma (ICP, Optima 7300DV & Avio500) spectrometer. Also, the surface-functionalizing elements of the Au LSPR strip, which are S and nitrogen (N), were analyzed by X-ray photoelectron spectroscopy (XPS, ThermoFisher).

## Results and Discussion

**Figure 1:**
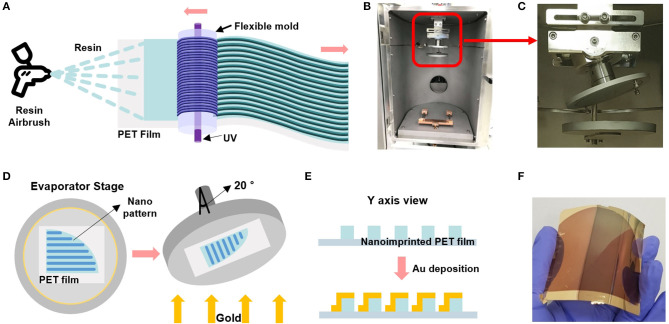
**(A)** Schematic of the roll-to-roll nanoimprinted strip fabrication with airbrushing-based resin coating to the PET film. **(B)** A digital camera image of the thermal evaporator chamber. **(C)** Magnified image of the red lined box in figure **(B)** shows a 20° tilted shaft of the wafer stage. **(D)** Schematic of the strip sample-equipped stage that is tilted by 20° to form the double-bent Au nanostructures. **(E)** Cross-sectional view of the double-bent Au-deposited PET film. **(F)** Gold-deposited flexible and transparent PET film.

To create the double-bent Au nanostructure, we thermally evaporated Au onto the nanograted PET film at 20° tilting angle. Figure 1 shows a schematic of roll-to-roll fabrication of nanolines on the PET film, upon which Ti/Au was tilted-deposited by thermal evaporator. Figure 1A describes the roll-to-roll imprinting process on the PET film. First, we wrapped a cylindrical roll with an imprinting mold for stamping nanoarchitecture on the PET film coated with UV-curable polymer resin using an airbrush coating process (Koo et al., 2016). As the resin-coated PET films pass through the cylindrical roller, UV light cures the imprinted film at the outlet. The digital camera image of Figure 1B shows a thermal evaporator chamber. The red lined box region of Figure 1B is magnified in Figure 1C. We tilted the wafer stage by 20° to produce a double-bent Au nanoarchitecture on one side of the nanoarchitecture wall. Figure 1D describes how to equip the nanoimprinted PET film on the wafer stage of the chamber in the thermal evaporator. The direction of the imprinted nanoline should be parallel to that of the tilting axis, as shown in the right picture of Figure 1D. Figure 1E is a schematic of a cross-sectional view before and after Au deposition upon the nanoimprinted PET film. Note that the tilted deposition of Au allows for double-bent Au structure which is an essential element for LSPR property. Figure 1F is a digital camera image of the double-bent Au-deposited PET film for the LSPR sensing platform.

The angled deposition of Au on the nanograting PET film has significant advantages. The conditions of the Au LSPR strip were tested by changing the deposition angles as shown in Figure S2. As a control sample, when Au was deposited on a flat PET film (without nanograting structures), we could nearly observe the extinction peak, as described in Figure S2A. However, as Au is deposited at various angles on the nanograting PET film, LSPR peaks were identified at different wavelengths, as shown in Figure S2B. We obtained the most pronounced LSPR peak at the tilting angle of 20°, hence we used this condition afterwards.

**Figure 2:**
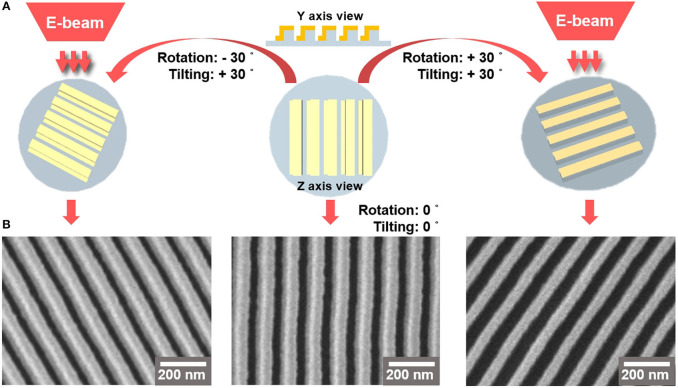
**(A)** 3D-schematics of SEM imaging of Au-deposited strips. **(B)** The left and right SEM images are Au-deposited nanoarchitecture films taken at rotating angle of 30° and tilting angle of 30°. The left and right images were obtained by rotating the stage counterclockwise and clockwise, respectively. The middle SEM image shows a top-down view of the Au LSPR strip.

To confirm the structure of the Au on the PET film, we performed SEM characterization by tilting-and-rotating the sample (Nam, 2018; Baek et al., 2020). Figure 2A describes the approach to visualizing the Au deposited on the side wall which is formed on the nanoimprinted PET film. We rotated the wafer stage either counterclockwise or clockwise by 30°, followed by 30° of tilting. Figure 2B shows that the SEM images of the nanoline wall sides are different, as the left side wall is deposited with Au (left image) while the right side wall is free from Au deposition (right image). The middle image of Figure 2B is a top-down image of the Au LSPR strip.

**Figure 3:**
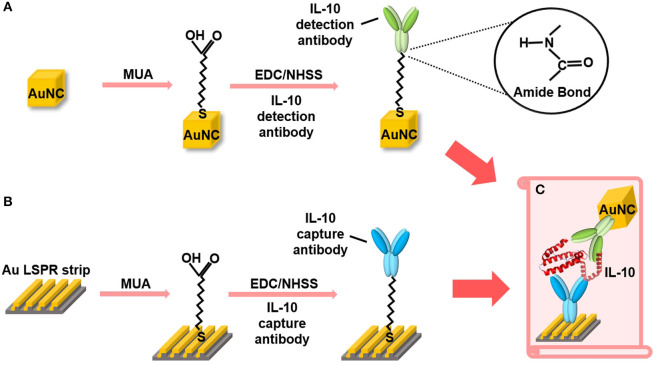
Schematic overview of the AuNC-assisted Au LSPR sensor strategy. **(A)** IL-10 detection antibody functionalization on the AuNC surfaces via the formation of a MUA monolayer followed by EDC/NHSS cross-linking chemistry. **(B)** The same chemistry was used for the immobilization of IL-10 capture antibody to the Au LSPR strip. **(C)** A dual Au LSPR sensor platform in which IL-10 cytokine is sandwiched by AuNC and Au strip.

Figure 3 provides an overview of the functionalization of AuNC and Au LSPR strip. The AuNC were synthesized as briefly described in Figure S1. We tested various shapes of Au nanoparticles including nanosphere, nanorod, and nanocube. Among them, the condition of AuNC is highly suitable for our experiments, since it allows for monodisperse colloidal structure and uniform plasmonic property as described in Figure S3. In addition, AuNC is well resuspended in buffer solution during stabilization of the coated antibody on the Au surface. The synthesized AuNC were characterized by TEM and EDX, as shown in Figure S3. For biofunctionalization, we treated the AuNC surface with MUA followed by EDC/NHSS cross-linking chemistry to form covalent attachment of IL-10 detection antibody as described in Figure 3A (Baek et al., 2014; Jang et al., 2014). Briefly, we added ethanolic MUA to the colloidal AuNC to form SAM on the AuNC surface. Next, we functionalized the colloidal AuNC using EDC/NHSS cross-linking chemistry, which is necessary for the formation of amide bonds between SAM and antibody, as described in the inset of Figure 3A.

Figure 3B shows the surface modification process of the Au LSPR strip. Similar to the functionalization of the colloidal AuNC, the double-bent Au structures were treated with MUA followed by EDC/NHSS cross-linking chemistry. The IL-10 capture antibody is then immobilized on the surface of the Au LSPR strip. Figure 3C describes the formation of a sandwich ELISA for IL-10 antigen by coupling the colloidal AuNC with the Au LSPR strip. The IL-10 capture antibody-coated Au LSPR strip is loaded with IL-10 antigen, followed by IL-10 detection antibody. The details of overall process including washing steps for the AuNC-conjugated Au LSPR strip assay are summarized in Figure S4.

**Figure 4:**
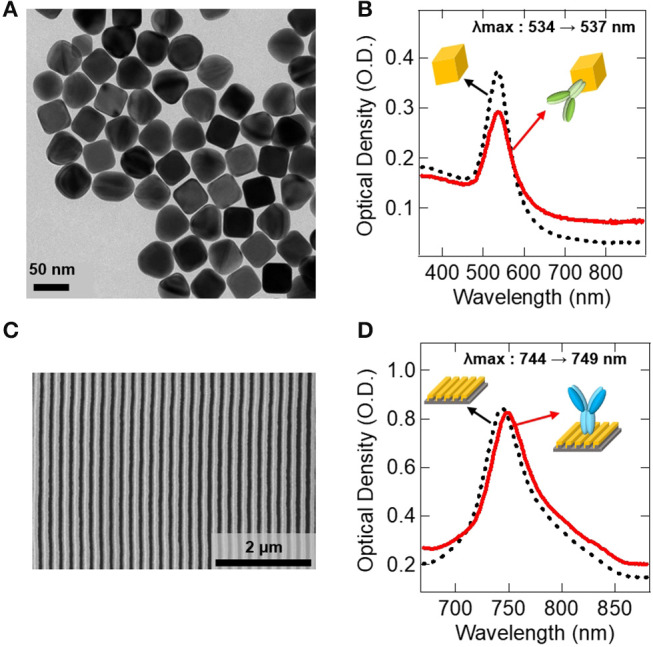
Antibody immobilization on the Au surfaces. **(A)** TEM image of the synthesized AuNC. **(B)** UV-vis spectrum data showing the immobilization of IL-10 detection antibody on the AuNC. **(C)** SEM image of the fabricated Au LSPR strip. **(D)** UV-vis spectrum data showing the immobilization of IL-10 capture antibody on the Au LSPR strip. In **(B,D)**, black dotted lines represent before antibody conjugation, and red lines are after antibody conjugation to Au surfaces.

Figure 4 shows the result of the surface functionalization of the AuNC and the Au LSPR strip. As shown in the TEM image in Figure 4A, the monodispersed AuNC has an extinction peak at 534 nm, as shown in Figure 4B. Figure 4C shows the SEM image of the Au LSPR strip, which has a strong extinction peak at 744 nm, as shown in Figure 4D. The shift of extinction peak was characterized before and after antibody immobilization on the colloidal AuNC and the Au LSPR strip, as shown in Figures 4B,D, respectively. The LSPR peak shift originates from the change of the refractive index around the surface of the Au structures. In this measurement, the peak of the AuNC shifts from 534 to 537 nm (Figure 4B), and the peak of the Au LSPR strip shifts from 744 to 749 nm (Figure 4D), indicating that biomolecules are immobilized on the Au surfaces. Therefore, the red shifts of the extinction peak were found to be 3 and 5 nm for AuNC and Au LSPR, respectively, which is in agreement with the previous findings (Hall et al., 2011). Red shifts are typically observed after antibody conjugation, which implies the increase in the dielectric constant around Ag or Au surfaces due to the additional molecular layers (Willet and Van Duyne, 2007). To confirm the immobilization of the antibody on the Au surface, we characterized the AuNC and Au LSPR strip surface by ICP and XPS, respectively. In Figure S5, ICP data of the colloidal AuNC shows the appearance of the S peak after MUA was treated. In Figure S6, XPS data of the surface of the Au LSPR strip present the appearance of S and N peaks after MUA and IL-10 antibody were treated, respectively. We observed the increased N signal implying that IL-10 antibody was successfully conjugated with the Au LSPR strip (Guo et al., 2017).

**Figure 5:**
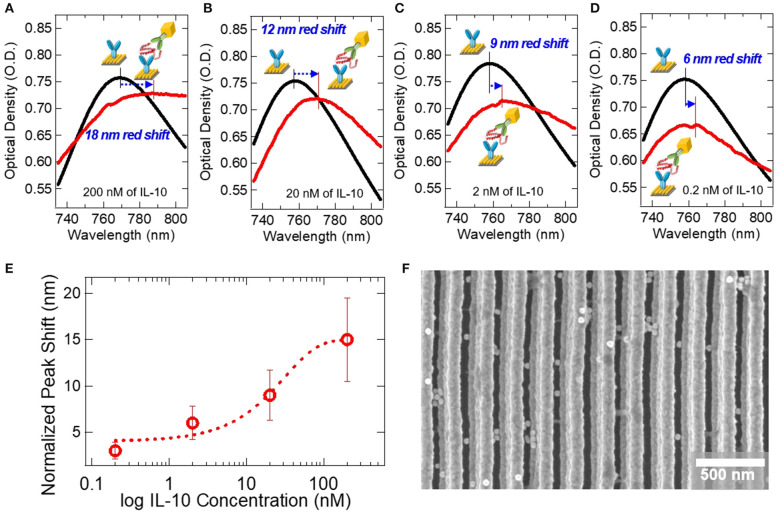
Extinction spectra for the detection of IL-10 on the AuNC-conjugated Au LSPR sandwich assay platform. Black lines represent extinction spectra of the IL-10 capture antibody-immobilized Au LSPR strip, and red lines are after the specific binding of IL-10 protein and IL-10 detection antibody. The concentrations of IL-10 were **(A)** 200 nM, **(B)** 20 nM, **(C)** 2 nM, and **(D)** 0.2 nM. **(E)** A plot of the extinction peak shift at varying IL-10 concentration from 0.2 to 200 nM. Error bars were calculated from an average of 3 repeat measurements. **(F)** SEM image of the AuNC-conjugated Au LSPR strip platform.

Figure 5 shows the extinction spectrum demonstrating the colloidal AuNC-based ELISA on the double-bent Au LSPR strip. We examined the effect of IL-10 concentration on the peak shift of extinction spectra, as shown in Figures 5A–D. In Figure 5A, the as-prepared LSPR chip shows UV spectrum curve with an extinction peak at ~760 nm. After soaking the LSPR chip in ELISA reagent solutions, we monitored the peak shift using bench-top microplate reader. Instead of conventional enzyme-substrate reactions such as horseradish peroxidase (HRP)-3,3′,5,5′-Tetramethylbenzidine (TMB) system (Van Weemen and Schuurs, 1971), we introduced the AuNC-conjugated detection antibody system as shown in the inset of Figure 3C. After completion of the reactions with IL-10 at concentrations of 200, 20, 2, and 0.2 nM followed by washing steps to remove excess AuNC as well as non-specific binding molecules, we obtained the red-colored spectra in Figures 5A–D, respectively. The detailed protocol is represented in Figure S4. When the 200 nM concentration of IL-10 protein was applied to the sandwich ELISA, the extinction peak was red-shifted by 18 nm from 769 to 787 nm as indicated by the blue arrow in Figure 5A.

At a lower IL-10 concentration of 20 nM, we monitored the extinction peak shift as shown in Figure 5B. After the ELISA reaction, the extinction peak is red-shifted from 758 to 770 nm, a peak shift of 12 nm. At even lower concentrations, such as 2 and 0.2 nM, as shown in Figures 5C,D, the peak shifts are measured as 9 and 6 nm, respectively. In Figure 5E, the extinction peak-shifts are plotted as a function of IL-10 concentration in the nanomolar range. SEM image in Figure 5F shows the AuNC-conjugated Au LSPR strip structure for the concentration of 200 nM IL-10 assay sample.

To evaluate non-specific adsorption, we designed four different types as negative controls, as summarized in Table 1 (e–h). For the first [Table 1 (e)] and second [Table 1 (f)] negative controls, IL-2 and BSA proteins were used instead of IL-10 at a concentration of 200 nM, respectively. For the third negative control [Table 1 (g)], we used only IL-10 detection antibody without AuNC conjugation. In Table 1 (a) and Table 1 (g), which are the same conditions except the presence of AuNC, the extinction peak shifts are 18 nm and 4 nm, respectively. Therefore, we estimate that the AuNC's amplification performance reaches to 450%. This suggests that plasmon coupling occurs between colloidal AuNC and Au LSPR strip. For the last negative control [Table 1 (h)], we used IL-6 detection antibody instead of IL-10 detection antibody. For all negative control samples, the extinction peak shift was less than 4 nm, which indicates that the contribution of nonspecific adsorption to the LSPR signal was negligible.

**Table 1:** Summary of the dual Au LSPR sensor platform and the negative controls (NCs).

	**(a)**	**(b)**	**(c)**	**(d)**	**(e) NC1**	**(f) NC2**	**(g) NC3**	**(h) NC4**
Protein	IL-10 200 nM	IL-10 20 nM	IL-10 2 nM	IL-10 0.2 nM	IL-2 200 nM	BSA	IL-10 200 nM	
Detection antibody	IL-10 detection antibody						No AuNC	IL-6 detection antibody
Scheme	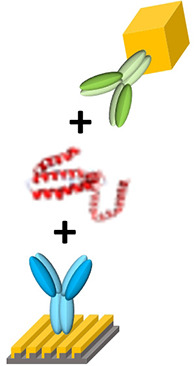				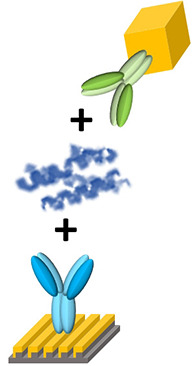	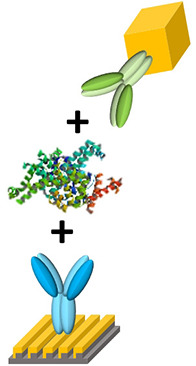	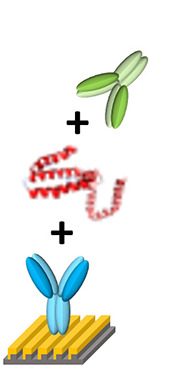	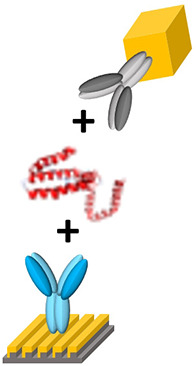
(i) AuNC peak	537	537	536	537	537	536	None	537
(ii) Antibody immobilized Au LSPR chip peak	769	758	758	758	755	756	749	754
(iii) After washing	787	770	767	764	758	758	753	757
(iii) - (ii)	18	12	9	6	3	2	4	3

## Conclusions

We report a dual Au LSPR assay platform that can detect IL-10 protein in the nanomolar range. We fabricated double-bent Au structures on PET film using roll-to-roll nanoimprinting to obtain an extinction peak at 759 (±10) nm. Using AuNC and Au LSPR strip, we successfully demonstrated the LSPR-based analytical strategy for IL-10 detection. The enhancement of extinction peak shift can be explained by a combination of two mechanisms: One is the increase of refractive index, and the other is plasmonic coupling between AuNC and Au LSPR strip. The multi-bent Au nanostructure has a strong plasma oscillation, yet the extinction peak shift is very small when the binding events occur only by the analytes and antibody proteins. By introducing the AuNC as an enhancing factor, we demonstrate the dual AuNC-Au LSPR strip assay which can be easily applied to the detection of various biomolecules with high sensitivity.

## Data Availability Statement

The raw data supporting the conclusions of this article will be made available by the authors, without undue reservation, to any qualified researcher.

## Author Contributions

S-WN, SB, and HL conceived the idea and designed the experiments. SB performed the nanoparticle synthesis, fabrication, assay preparation, and optical characterization. SB and S-WN performed electron microscopy, spectroscopic, and spectrometric analysis. HS and MK conducted the fabrication of nanoimprinted films. SL assisted with evaporator operation. J-EK and YK suggested the model protein. MK, J-SW, and JO provided the nanoimprinted films. JP and S-WN acquired funding. S-WN, SB, and HL wrote the manuscript. SB, MK, HL, and S-WN discussed the results, and all authors provided comments on the manuscript.

## Conflict of Interest

The authors declare that the research was conducted in the absence of any commercial or financial relationships that could be construed as a potential conflict of interest.

## References

[B1] Baek S. H. Lee S. Bae J-H. Hong C-W. Park M-J. Park H. (2020). Nanopillar and nanohole fabrication via mixed lithography. Materials Research Express 7, 035008 10.1088/2053-1591/ab77ed

[B2] Baek S. H. Wark A. W. Lee H. J. (2014). Dual nanoparticle amplified surface plasmon resonance detection of thrombin at subattomolar concentrations. Anal. Chem. 86, 9824–9829. 10.1021/ac5024183 25186782

[B3] Bellassai N. D'Agata R. Jungbluth V. Spoto G. (2019). Surface plasmon resonance for biomarker detection: Advances in non-invasive cancer diagnosis. Front. Chem. 7:570. 10.3389/fchem.2019.00570 31448267 PMC6695566

[B4] Chen S. Svedendahl M. Van Duyne R. P. Käll M. (2011). Plasmon-enhanced colorimetric ELISA with single molecule sensitivity. Nano Lett. 11, 1826–1830. 10.1021/nl2006092 21428275

[B5] Guo L. Yang Z. Zhi S. Feng Z. Lei C. Zhou Y. (2017). Sensitive detection of cardiac troponin T based on superparamagnetic bead-labels using a flexible micro-fluxgate sensor. RSC Adv. 7, 52327–52336. 10.1039/c7ra10355g

[B6] Hall W. P. Ngatia S. N. Van Duyne R. P. (2011). LSPR biosensor signal enhancement using nanoparticle-antibody conjugates. J. Phys. Chem. 115, 1410–1414. 10.1021/jp106912p 21660207 PMC3109750

[B7] Holzinger M. Goff A. L. Cosnier S. (2014). Nanomaterials for biosensing applications: a review. Front. Chem. 2:63. 10.3389/fchem.2014.00063 25221775 PMC4145256

[B8] Im H. Shao H. Park Y. I. Peterson V. M. Castro C. M. Weissleder R.. (2014). Label-free detection and molecular profiling of exosomes with a nano-plasmonic sensor. Nat. Biotechnol. 32, 490–495. 10.1038/nbt.2886 24752081 PMC4356947

[B9] Jang H. R. Wark A. W. Baek S. H. Chung B. H. Lee H. J. (2014). Ultrasensitive and ultrawide range detection of a cardiac biomarker on a surface plasmon resonance platform. Anal. Chem. 86, 814–819. 10.1021/ac4033565 24328254

[B10] Koo S. Lee S. H. Kim J. D. Kim H.ong J. G. Baac H. W. Kwak M. K. (2016). Controlled airbrush coating of polymer resists in roll-to-roll nanoimprinting with regimented residual layer thickness. Int. J. Precis. Eng. Manuf. 17, 943–947. 10.1007/s12541-016-0115-8

[B11] Liu B. Huang R. Yu Y. Su R. Qi W. He Z. (2018). Gold nanoparticle-aptamer-based LSPR sensing of ochratoxin A at a widened detection range by double calibration curve method. Front. Chem. 6:94. 10.3389/fchem.2018.00094 29670875 PMC5893832

[B12] Mayer K. M. Hafner J. H. (2011). Localized surface plasmon resonance sensors. Chem. Rev. 111, 3828–3857. 10.1021/cr100313v 21648956

[B13] McFarland A. D. Van Duyne R. P. (2003). Single silver nanoparticles as real-time optical sensors with zeptomole sensitivity. Nano Lett. 3, 1057–1062. 10.1021/nl034372s

[B14] Miller M. M. Lazarides A. A. (2005). Sensitivity of metal nanoparticle surface plasmon resonance to the dielectric environment. J. Phys. Chem. B. 109, 21556–21565. 10.1021/jp054227y 16853799

[B15] Nam S-W. (2018). 200 mm wafer-scale fabrication of polydimethylsiloxane fluidic devices for fluorescence imaging of single DNA molecules. MRS Communications 8, 420–427. 10.1557/mrc.2018.58

[B16] Sau T. K. Murphy C. J. (2004). Room temperature, high-yield synthesis of multiple shapes of gold nanoparticles in aqueous solution. J. Am. Chem. Soc. 126, 8648–8649. 10.1021/ja047846d 15250706

[B17] Sepúlveda B. Angelomé P. C. Lechuga L. M. Liz-Marzán L. M. (2009). LSPR-based nanobiosensors. Nano Today 4, 244–251. 10.1016/j.nantod.2009.04.001

[B18] Sönnichsen C. Reinhard B. M. Liphardt J. Alivisatos A. P. (2005). A molecular ruler based on plasmon coupling of single gold and silver nanoparticles. Nat. Biotechnol. 23, 741–745. 10.1038/nbt1100 15908940

[B19] Storhoff J. J. Lucas A. D. Garimella V. Bao Y. P. Müller U. R. (2004). Homogeneous detection of unamplified genomic DNA sequences based on colorimetric scatter of gold nanoparticle probes. Nat. Biotechnol. 22, 883–887. 10.1038/nbt977 15170215 PMC1201475

[B20] Van Weemen B. K. Schuurs A. H. W. M. (1971). Immunoassay using antigen-enzyme conjugates. FEBS Lett. 15, 232–236. 10.1016/0014-5793(71)80319-8 11945853

[B21] Wang S.-S. Zhao X-P. Liu F-F. Younis M. R. Xia X-H. Wang C. (2019). Direct plasmon-enhanced electrochemistry for enabling ultrasensitive and label-free detection of circulating tumor cells in blood. Anal. Chem. 91, 4413–4420. 10.1021/acs.analchem.8b04908 30816698

[B22] Wi J-S. Lee S. Lee S. H. Oh D. K. Lee K-T. Park I.. (2017). Facile three-dimensional nanoarchitecturing of double-bent gold strips on roll-to-roll nanoimprinted transparent nanogratings for flexible and scalable plasmonic sensors. Nanoscale 9, 1398–1402. 10.1039/C6NR08387K 28070589

[B23] Willet K. A. Van Duyne R. P. (2007). Localized surface plasmon resonance spectroscopy and sensing. Annu. Rev. Phys. Chem. 58, 267–297. 10.1146/annurev.physchem.58.032806.104607 17067281

[B24] Wu X. Ming T. Wang X. Wang P. Wang J. Chen J. (2010). High-photoluminescence-yield gold nanocubes: for cell imaging and photothermal therapy. ACS Nano 4, 113–120. 10.1021/nn901064m 20014823

[B25] Xu D. Yu S. Yin Y. Wang S. Lin Q. Yuan Z. (2018). Sensitive colorimetric Hg^2+^ detection via amalgamation-mediated shape transition of gold nanostars. Front. Chem. 6:566. 10.3389/fchem.2018.00566 30538981 PMC6277514

