# Supplementary material for: Gold Nanoparticle-Enhanced and Roll-to-Roll Nanoimprinted LSPR Platform for Detecting Interleukin-10
Source: Front Chem. 2020 May 26;8:285. doi: 10.3389/fchem.2020.00285 (PMC7264386; doi:10.3389/fchem.2020.00285)
Supplement: Supplementary file 1 [file Data_Sheet_1.docx]

***Supplementary Material***

**Au Nanoparticle-Enhanced Roll-to-Roll Nanoimprinted Localized Surface Plasmon Resonance Platform for Detecting Interleukin-10 Cytokine**

Seung Hee Baek^1^, Hyun Woo Song^2^, Sunwoong Lee^1^, Jung-Eun Kim^1^, Yeo Hyang Kim^3^, Jung-Sub Wi^4^, Jong G. Ok^5^, Jun Seok Park^6^, Seonki Hong^7^, Moon Kyu Kwak^2^*, Hye Jin Lee^8^*, and Sung-Wook Nam^1^*

^1^Department of Molecular Medicine, School of Medicine, Kyungpook National University, Daegu 41405, South Korea

^2^Department of Mechanical Engineering, School of Mechanical Engineering, Kyungpook National University, Daegu 41566, South Korea

^3^Department of Pediatrics, School of Medicine, Kyungpook National University, Daegu 41404, South Korea

^4^Center for Nano-Bio Measurement, Korea Research Institute of Standards and Science, Daejeon 34113, South Korea

^5^Department of Mechanical and Automotive Engineering, Seoul National University of Science and Technology, Seoul 01811, South Korea

^6^Department of Surgery, School of Medicine, Kyungpook National University, Daegu 41404, South Korea

^7^Department of Emerging Materials Science, Daegu Gyeongbuk Institute of Science and Technology (DGIST), Daegu 42988, South Korea

^8^Department of Chemistry and Green Nano Materials Research Center, Kyungpook National University, Daegu 41566, South Korea

*Correspondence: M.K.K. ([mkkwak@knu.ac.kr](mailto:mkkwak@knu.ac.kr)), H.J.L. ([hyejinlee@knu.ac.kr](mailto:hyejinlee@knu.ac.kr)), and S.-W.N. ([nams@knu.ac.kr](mailto:nams@knu.ac.kr))


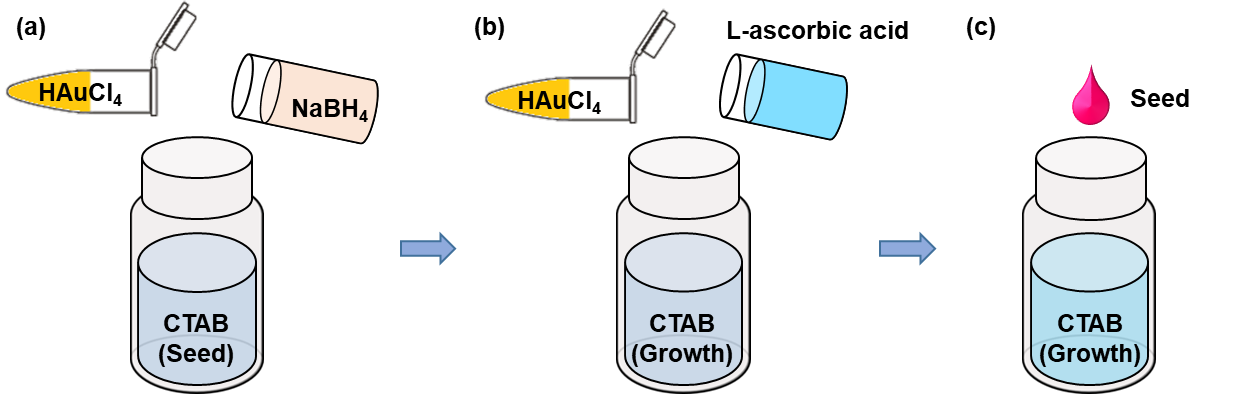


**Figure S1.** Schematic of Au nanocube (AuNC) synthesis. (a) The seed nanoparticles were prepared by adding gold (III) chloride hydrate (HAuCl_4_) and sodium borohydride (NaBH_4_) to hexadecyltrimethylammonium bromide (CTAB) solution. (b) HAuCl_4_ and L-ascorbic acid were consecutively added to the CTAB solution to prepare the growth solution. (c) The 10-fold diluted seed solution was added to the growth solution. The resulting solution was reacted overnight at 29 °C.


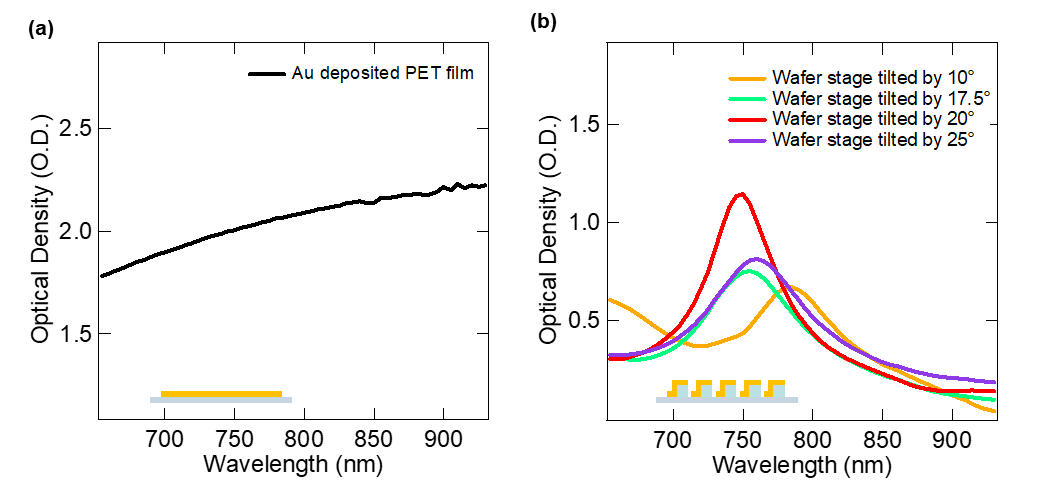


Figure S2. Comparison of localized surface plasmon resonance (LSPR) peaks with various tilting angles of the wafer stage for gold (Au) deposition. (a) Ultraviolet-visible spectroscopy data of Au deposited polyethylene terephthalate film without nanograting structure. (b) The orange, green, red and purple lines represent LSPR peaks observed when nanograting structures are installed at different inclined angles of 10°, 17.5°, 20° and 25° in the thermal evaporator, respectively.


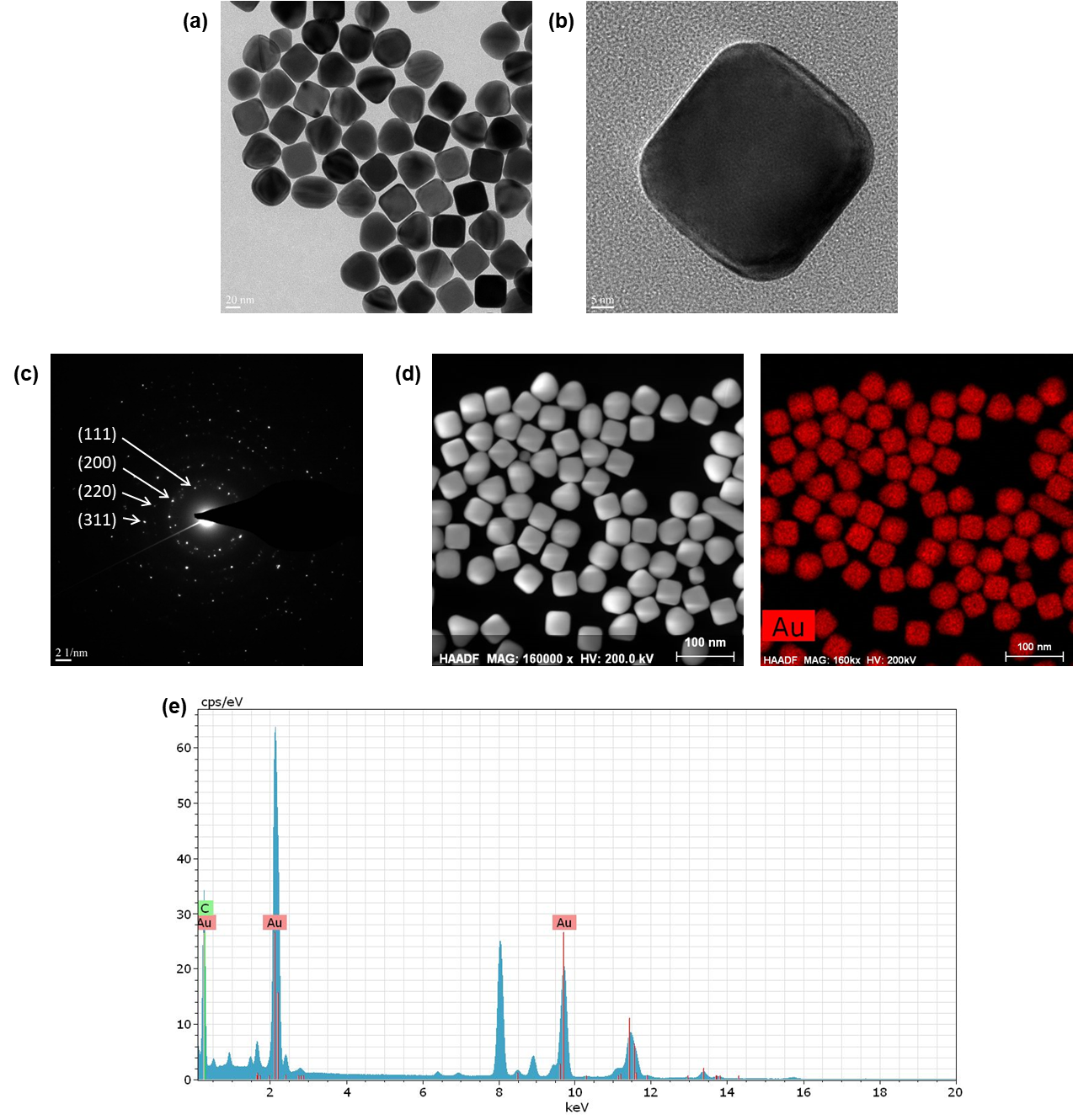


**Figure S3.** Transmission electron microscopy (TEM) characterization of AuNC. (a) TEM image of monodispersed AuNC. (b) High-resolution image of a single AuNC. (c) Diffraction pattern image. (d) Scanning transmission electron microscopy and energy-dispersive X-ray spectroscopy (EDX) mapping images. (e) EDX spectrum of AuNC.


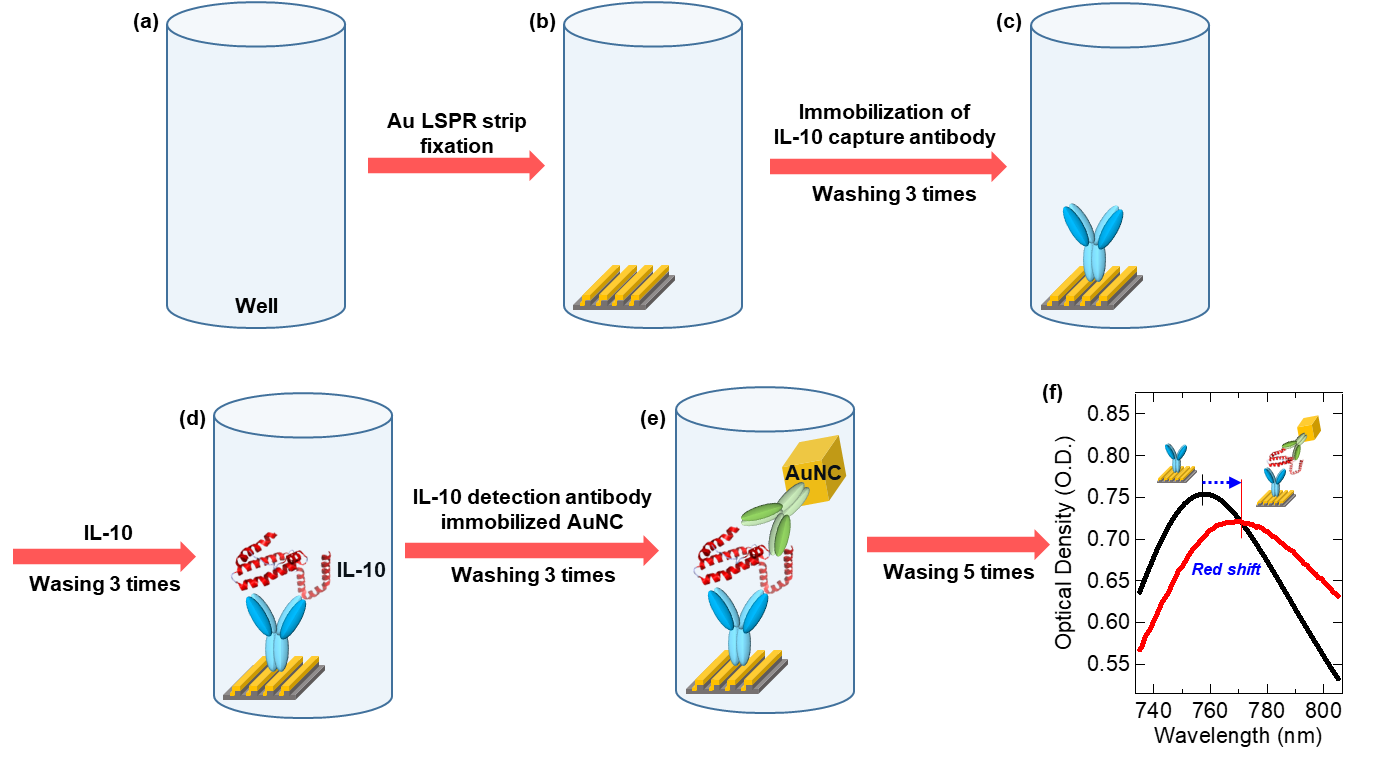


**Figure S4.** Overview of the dual-Au LSPR sandwich assay platform. (a) A well of a 96-well plate. (b) The Au LSPR strip sample with a 5-mm diameter is physically attached to the bottom of the 96-well plate. (c) Interlukin-10 (IL-10) capture antibody is immobilized on the Au LSPR strip using 1-ethyl-3-(3-dimethylaminopropyl) carbodiimide hydrochloride (EDC) and N-hydroxysulfosuccinimide (NHSS) cross-linking chemistry. (d) IL-10 specifically binds to the IL-10 capture antibody. (e) IL-10 detection antibody was immobilized to the surface of AuNC. The AuNC conjugated with the detection antibody is introduced for specific adsorption with IL-10. (f) Representative extinction spectra. For each step of biomolecular binding, the Au LSPR strips were washed at least 3~5 times to remove non-specific absorption.

**
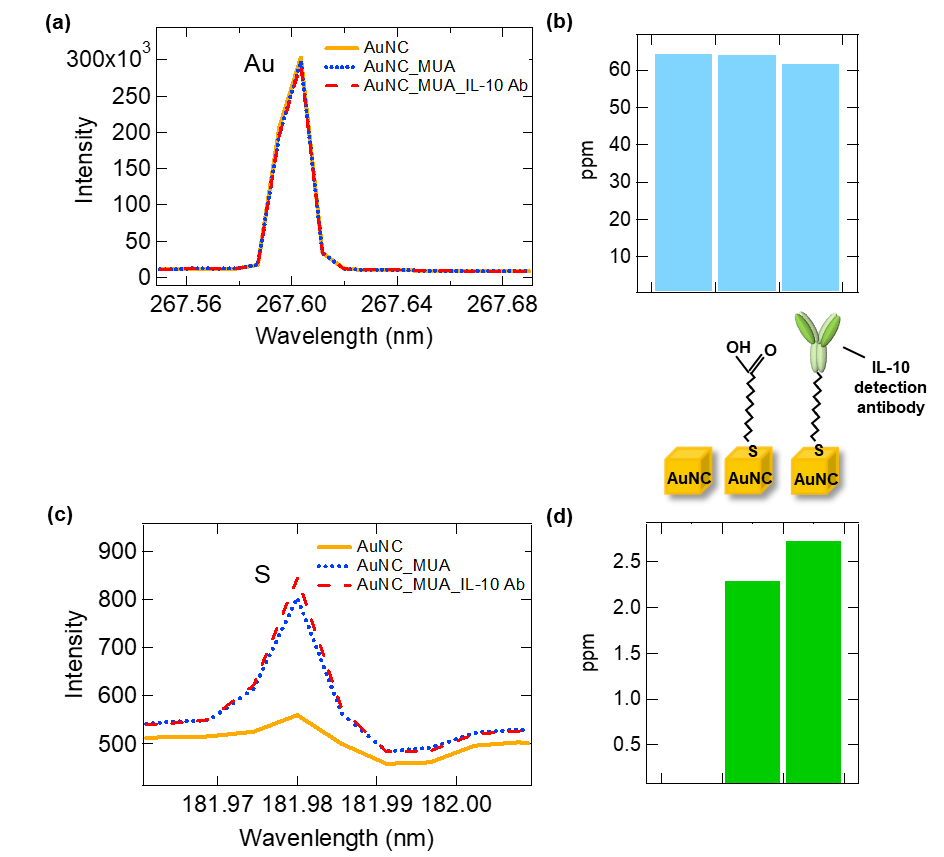
**

**Figure S5.** Inductively coupled plasma (ICP) spectrometers of colloidal AuNC. (a) The peaks of Au for the pristine AuNC (orange line), 11-mercaptoundecanoic acid (MUA) treated AuNC (blue line), and IL-10 antibody coated-MUA-treated AuNC (red line). (b) The concentration of Au measured in ICP spectrometer, as the surface of the AuNC is modified by MUA followed by IL-10 antibody. (c) The peaks of sulfur (S) for the surface-modified AuNCs. (d) The concentration of S is increased as the AuNC is treated by MUA.


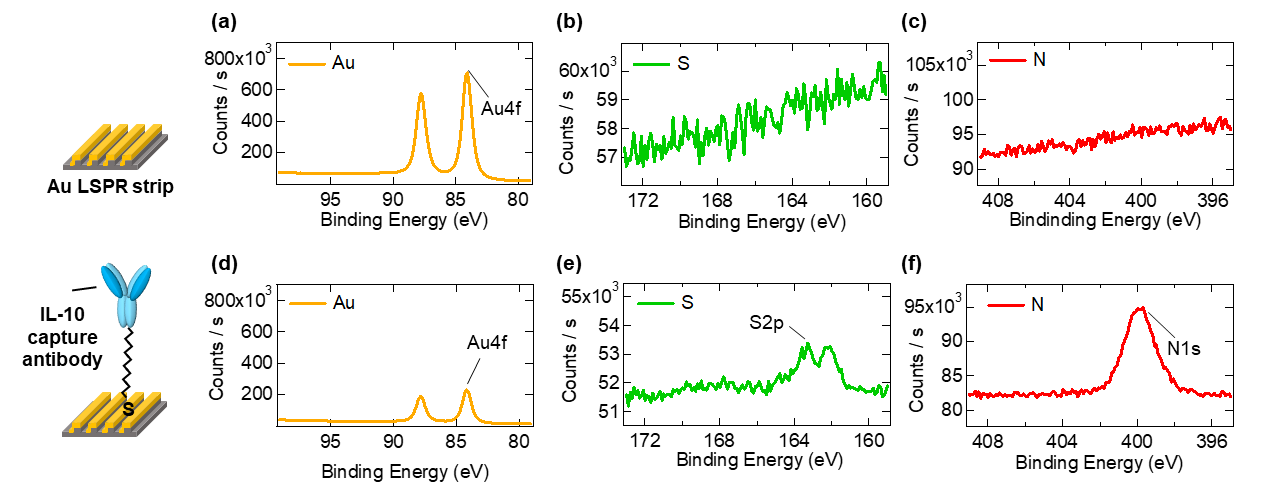


**Figure S6.** X-ray photoelectron spectroscopy (XPS) analysis of the surface-functionalized Au LSPR strip. (a,b,c) Au, S, and nitrogen (N) peaks of the pristine Au LSPR strip after Au is evaporated on the nanograting pattern. (d,e,f) Au, S, and N peaks of the MUA treated Au LSPR strip followed by IL-10 antibody immobilization.
